# Next-generation anti-infective drugs in the post-antibiotic era: focusing on anti-virulence agents targeting the bacterial quorum sensing system

**DOI:** 10.3389/fcimb.2026.1935576

**Published:** 2026-08-20

**Authors:** Xin Hu, Yang Yuan, Zhiyong Yang, Yiwen Chu, Ting Huang, Kelei Zhao

**Affiliations:** 1 Clinical Medical College & Affiliated Hospital of Chengdu University, Chengdu University, Chengdu, China; 2 Antibiotics Innovation and Resistance Control Key Laboratory of Sichuan Province, School of Pharmacy, Chengdu University, Chengdu, China

**Keywords:** alternative therapeutic, antimicrobial resistance, anti-virulence agents, quorum sensing, quorum sensing inhibitors

## Abstract

Antimicrobial resistance is a critical global public health challenge, with drug-resistant infections contributing to more than one million deaths annually. The widespread dissemination of multidrug-resistant bacteria poses a severe threat to the management of infectious diseases. Bacterial evolution via genetic mutation and horizontal gene transfer diminishes antimicrobial efficacy, often leading to therapeutic failure, increased morbidity and mortality. However, the development of novel antibiotics lags far behind the rapid evolution of drug-resistant bacteria. Therefore, scientists worldwide have committed to exploring alternative therapeutic strategies for bacterial infections. The key question is which strategy holds the greatest promise of addressing the predicament of traditional antibiotics and being recognized as “next-generation anti-infective drugs”. This narrative review summarizes several of the most promising alternative treatment strategies against bacterial infections, emphasizing the core strengths and limitations of each strategy. A critical comparative analysis reveals that no single strategy can simultaneously satisfy the demands of acute therapy, broad patient coverage, and resistance evasion, underscoring the need for context-dependent and sequential deployment. Moreover, among these alternatives, anti-virulence therapeutic strategies, particularly those targeting the bacterial quorum sensing (QS) system, represent a major and extensively studied approach, although their clinical translation remains nascent. This review delineates the molecular mechanisms and therapeutic potential of QS-targeting anti-virulence agents. Furthermore, we candidly assess the extant biological, pharmacological, and clinical barriers impeding their clinical translation, providing perspectives on future research directions to harness these next-generation anti-infective paradigms effectively.

## 1 Introduction

The spread of multidrug-resistant bacteria has made antimicrobial resistance (AMR) one of the most critical global public health challenges (Theuretzbacher et al., 2020; Larsson and Flach, 2022). AMR has been detected in nearly all clinically relevant bacterial strains, pushing the world toward a post-antibiotic era (Whiteley et al., 2017). Infections triggered by drug-resistant pathogens pose substantial clinical challenges and are linked to poor clinical prognoses, such as increased morbidity and mortality (Nadeem et al., 2020). Between 1990 and 2021, drug-resistant infections contributed to more than one million deaths annually. Projections indicate that by 2050, this figure could reach 10 million fatalities per year. AMR exacerbates the burden of infectious diseases. However, improving access to antibiotics and optimizing treatment strategies could save up to 92 million lives between 2025 and 2050—compared with current trajectories (Naddaf, 2024).

Traditional antibiotics pose significant challenges for infection treatment. By directly killing or inhibiting bacterial growth to treat infections, they exert a strong selective pressure on bacterial survival, readily selecting for resistance mutations and, in some cases, promoting the horizontal transfer of resistance genes (Daruka et al., 2025). Long-term use substantially impacts the host’s commensal microbial communities, often leading to dysbiosis and secondary infections, such as *Clostridium difficile* infections (Leffler and Lamont, 2015). They also fail to meet the needs of patients with chronic infections, such as *Pseudomonas aeruginosa* colonization in cystic fibrosis, where antibiotics are often ineffective at eradicating biofilms, and repeated administration accelerates the evolution of resistance (Song et al., 2021; Grasemann and Ratjen, 2023). However, developing new antibiotics is both time-consuming and costly, and their clinical utility is often short-lived, as resistance can emerge within a relatively brief period after their introduction (Spagnolo et al., 2021; Garlasco et al., 2026). Meanwhile, the global overuse and misuse of antibiotics remain unchecked (Theuretzbacher, 2025). Therefore, combating antibiotic resistance requires a broad and innovative treatment strategy that extends beyond the use of traditional antibiotics.

**Figure 1:**
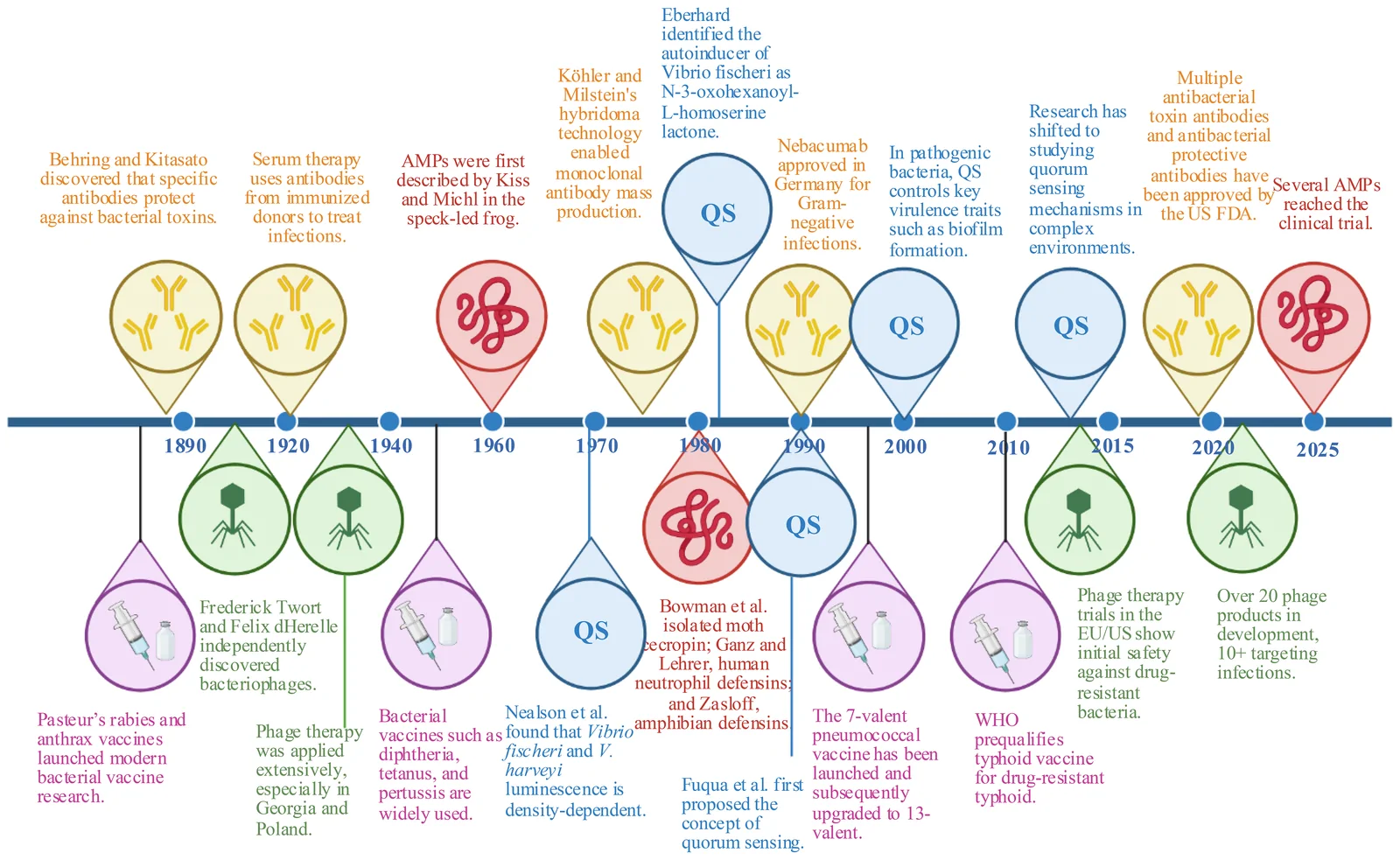
Timeline of alternative strategies against drug-resistant bacteria. This timeline highlights key milestones across major therapeutic approaches for drug-resistant bacterial infections. Purple: vaccines; yellow: antibody-based therapies; green: bacteriophages; red: antimicrobial peptides (AMPs); blue: anti-virulence agents targeting quorum sensing (QS). Created in BioRender (https://BioRender.com/jflayfr).

Notably, scientists worldwide have committed to exploring alternative therapeutic strategies for bacterial infections. Given this complex landscape, a pivotal comparative question merits careful consideration: which therapeutic modality can most effectively circumvent the inherent limitations of conventional antibiotics and rightfully be regarded as a “next-generation anti-infective”? Although the search for alternative antimicrobial strategies has persisted for over a century, numerous alternative approaches have been developed, as shown in **Figure 1**. Among alternative strategies, anti-virulence therapeutic strategies represent a highly promising novel approach to antimicrobial therapy that has garnered significant attention in recent years (Li et al., 2024). This alternative therapeutic strategy involves inactivating critical bacterial virulence factors or disrupting the intercellular signaling pathways responsible for producing these factors (Hibbert et al., 2024). Quorum sensing (QS) systems occupy a central hub within the complex regulatory networks that govern bacterial virulence, coordinating the expression of multiple virulence factors (Defoirdt, 2018; Wu et al., 2021). Consequently, this system is regarded as an ideal target for anti-virulence strategies and has become one of the most extensively studied targets in current research (Moreno-Gámez et al., 2023).

In this narrative review, we summarize several of the most promising alternative treatment strategies against bacterial infections, emphasizing the core strengths and limitations of each approach while comprehensively summarizing the latest research advances. This review provides a clear and panoramic view of the research in this field. Moreover, we focus on anti-virulence agents targeting bacterial QS systems, discuss their current research status, and critically assess their application potential and the challenges that lie ahead.

## 2 Categorizing alternative anti-infective strategies: a tripartite functional framework

**Figure 2:**
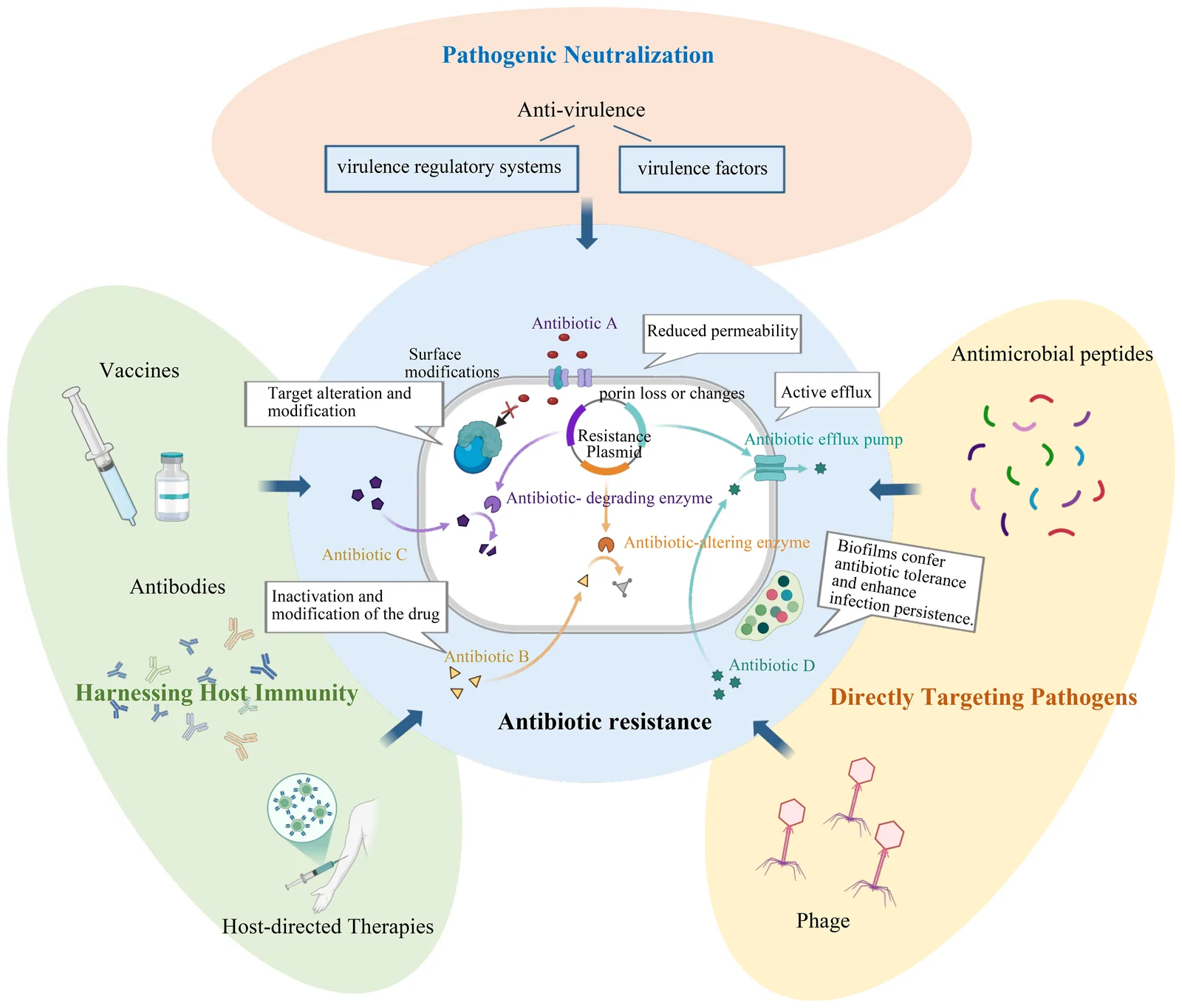
A functional classification of emerging alternative strategies against antibiotic-resistant bacterial infections. The peripheral segments of the figure illustrate the tripartite framework proposed in this review, categorizing alternative anti-infective approaches into three axes based on their mechanisms of action: (I) harnessing host factors: vaccines, therapeutic antibodies and host-directed therapies; (II) directly targeting pathogens (phage therapy and antimicrobial peptides); and (III) neutralizing bacterial virulence (anti-virulence strategies). The central segment summarizes the major resistance mechanisms deployed by bacteria against conventional antibiotics. These mechanisms include: reduced membrane permeability limiting drug uptake; target modification or protection preventing antibiotic recognition; enzymatic degradation or modification of antibiotics; active efflux via efflux pumps; and biofilm-mediated tolerance and persistence. These alternative strategies collectively aim to reduce selective pressure, preserve the host microbiome, and offer a more sustainable paradigm for combating bacterial infections, independent of traditional antibiotic mechanisms. Created in BioRender (https://BioRender.com/hr8e2ye).

As the crisis of AMR deepens, the traditional model of antibiotic research is reaching its limits. While numerous alternative strategies have been proposed, a critical examination reveals that they are often discussed in isolation, obscuring their inherent trade-offs. To address this challenge, we propose a functional classification of emerging therapies into three axes: harnessing host factors, direct pathogen elimination, and virulence neutralization, as illustrated in **Figure 2**.

### 2.1 Harnessing host factors: vaccines, therapeutic antibodies and host-directed therapies

This axis encompasses strategies that engage host elements to counteract infection, which can be broadly divided into immune-based interventions (vaccines and antibodies that rely on pre-existing or inducible immune components) and host cellular pathway modulators (host-directed therapies that regulate metabolism, autophagy, or inflammatory cascades). Vaccination may become an increasingly important strategy to prevent infections, reduce antibiotic use, and curb the spread of drug resistance (Poland and Kennedy, 2022; Huang et al., 2025). High vaccine coverage induces herd immunity, curbing pathogen transmission and the development of drug-resistant strains (Michie, 2022). Vaccines can trigger specific immune responses in early infection stages (Antonelli et al., 2021; Hou et al., 2023) and provide long-term protection, reducing recurrent infections (Cross, 2023; Miao et al., 2023). However, the development process is protracted, often spanning 10 to 20 years. Clinical efficacy trials for drug-resistant bacterial vaccines are particularly challenging because of low infection rates in target populations and difficulty in defining clinical endpoints (Aernout et al., 2025). The design of broad-spectrum vaccines for Gram-negative bacteria is complicated by numerous serotypes and antigenic variations (Henrique et al., 2022), and vaccine escape mutations have been documented (Frost et al., 2023; Peletta et al., 2023). For clinically prevalent drug-resistant bacteria such as *S. aureus*, *P. aeruginosa*, *K. pneumoniae*, and *E. coli*, no vaccines have been formally approved. Despite advanced candidates, including the OprF-OprI fusion protein (IC43) (Adlbrecht et al., 2020), the octavalent conjugate vaccine (Aerugen^®^) (Sainz-Mejías et al., 2020), and the bivalent flagellin vaccine for *P. aeruginosa* (Döring et al., 2007), all ultimately failed in Phase III trials.

Therapeutic antibodies, particularly monoclonal antibodies (mAbs), feature high specificity, minimal side effects, and a high success rate in development (Wang et al., 2022). They exert effects through toxin neutralization, complement-mediated lysis, and effector cell-mediated phagocytosis (Lu et al., 2020; Carter and Rajpal, 2022). Although mAbs have a narrow spectrum, the likelihood of resistance is low when the target antigen is highly conserved (Irani et al., 2015). However, the application of mAbs in bacterial infections is severely constrained by the limited access of IgG to infection sites, especially in abscesses or across certain physiological barriers (Simonis et al., 2023). Modifications such as glycosylation alterations and bispecific designs may partially overcome these limitations, but production remains expensive and scaling up manufacturing is challenging (Chang et al., 2022; Simonis et al., 2023). Currently, only a handful of mAbs are approved for bacterial infections, including Bezlotoxumab for *C. difficile* recurrence (Wang et al., 2022), while others like Tosatoxumab (anti-*S. aureus* α-toxin) (Piscaglia et al., 2025) and Gremubamab (bispecific anti-*P. aeruginosa*) (Chastre et al., 2022) remain in clinical trials.

Beyond specific immune induction, an alternative paradigm has gained momentum: host-directed therapies (HDTs), which aim to recalibrate the host cellular microenvironment rather than directly engaging the pathogen (Rastogi and Chandra, 2024; Zheng et al., 2025). Instead of targeting the pathogen, HDTs modulate host pathways exploited by bacteria for survival, immune evasion, and persistence (Kaufmann et al., 2018). Approaches include small molecules, polypeptides, and repurposed drugs that enhance immunity, regulate inflammation, or interfere with autophagy and metabolism (Mohan and Bhattacharya, 2020). Autophagy modulation (inhibition or activation) can restrict nutrient acquisition or enhance degradation (Zheng et al., 2025). However, HDTs face challenges such as context-dependent efficacy, off-target toxicity, host genetic variability, and precise dosing (Chiang et al., 2018). Although most candidates are preclinical, several have entered trials, with tuberculosis as the prime example; metformin and CC11050 (dovramilast) are in a Phase II RCT for rifampicin-resistant TB (Tukvadze et al., 2025). Many HDT candidates are repurposed drugs with verified safety, e.g., aspirin, ibuprofen, thalidomide, imatinib (Zumla et al., 2015), and clemizole (Yu et al., 2026). Novel compounds like KL (Lu et al., 2025) and indolocarbazoles (SB218078, GW296115X) (van den Biggelaar et al., 2025) show activity against intracellular *S. aureus* in preclinical models. While none are yet approved for bacterial infections, as adjuncts they hold potential to reduce selective pressure and pathology.

The fundamental paradox of all the above immune-based strategies lies in their temporal and populational mismatch. Vaccines require extended lead times, conflicting with the acute onset of severe infections, while mAbs, though faster, still depend on intact Fc receptor function. Their efficacy inherently declines in the very populations most vulnerable to AMR infections, namely the elderly, neonates, and immunocompromised patients (Dubé et al., 2021; Simonis et al., 2023). The efficacy of HDTs is highly contingent upon both the stage of infection and the host’s immune status, rendering the therapeutic window exceedingly narrow in acute, life-threatening infections (Hotchkiss and Monneret, 2026). This dependency renders them a complementary, rather than a foundational, solution to the AMR crisis.

### 2.2 Directly targeting pathogens: phage therapy and antimicrobial peptides

In contrast to immune reliance, phage therapy and antimicrobial peptides (AMPs) inflict direct damage on bacterial cells, acting independently of the host immune status. Phages target and kill specific host bacteria through bacteriolysis (Dion et al., 2020; Strathdee et al., 2023), showing potential in treating chronic and biofilm-associated infections (Chevallereau et al., 2022; Hatfull et al., 2022; Uyttebroek et al., 2022). Advantages include high specificity, self-amplification, ease of production, low intrinsic toxicity, and biofilm degradation (Schwarz et al., 2022; Peng et al., 2025). However, the narrow host range and rapid induction of bacterial resistance (as short as 15 to 30 minutes for single-phage treatments) critically limit their broad-spectrum use (Tal et al., 2021; McKitterick and Bernhardt, 2022; Stokar-Avihail et al., 2023). Prolonged or repeated administration can trigger immune responses that compromise efficacy, and large-scale randomized controlled trials confirming safety are insufficient (Uyttebroek et al., 2022; Yuan et al., 2023). Although engineered cocktails like Entelli-O2 (against *E. cloacae*) and AB_CK2 (against *A. baumannii*) have shown promise in animal models and compassionate use (Liu et al., 2025; Subedi et al., 2025), production standardization for personalized therapies remains a significant regulatory hurdle (Luong et al., 2020).

AMPs, or host defense peptides (Mahlapuu et al., 2020), exhibit rapid bactericidal action through membrane disruption and biofilm penetration (Mhlongo et al., 2023; MacNair et al., 2024). They target multiple pathways, complicating the development of single-step resistance (van der Does et al., 2019; Lazzaro et al., 2020; Mookherjee et al., 2020). However, systemic administration is challenged by poor pharmacokinetics, proteolytic degradation, serum neutralization, and potential cytotoxicity at high concentrations (Yeung et al., 2011; Magana et al., 2020; Mahlapuu et al., 2020). Cross-resistance risks also exist if AMPs resemble endogenous peptides. Although over 3,000 natural AMPs have been identified (Mahlapuu et al., 2020) and some have entered trials (e.g., NVB-302 for *C. difficile*) (Mookherjee et al., 2020), numerous candidates have failed due to insufficient *in vivo* efficacy or toxicity (Lazzaro et al., 2020).

Direct killing strategies face an evolutionary arms race that inherently favors the pathogen. Phages impose strong selective sweeps, rapidly enriching resistant mutants, while AMPs suffer from physiological instability that decouples promising *in vitro* activity from disappointing *in vivo* outcomes. Consequently, these strategies, while effective in specific niches, are inherently fragile when deployed as systemic monotherapies due to the sheer adaptability of bacterial populations and the hostile proteolytic environment of the human body.

### 2.3 Pathogenic neutralization: anti-virulence strategies

**Figure 3:**
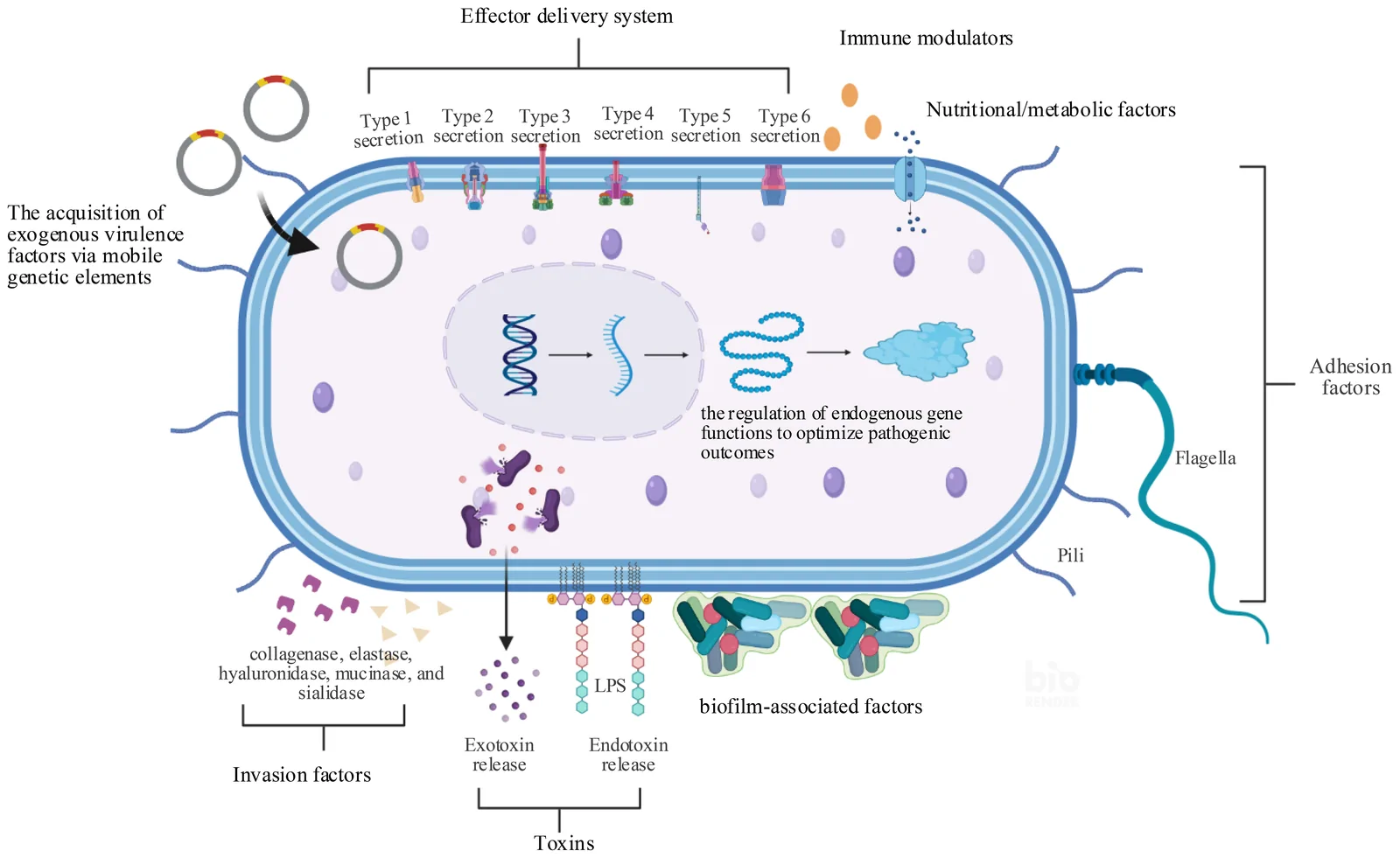
Bacterial virulence factors: classification and evolution. virulence factors are traditionally categorized into several groups, such as adhesion factors, invasion factors, toxins, effector delivery systems, immune modulators, biofilm-associated factors, and nutritional or metabolic factors, etc. The evolutionary success of pathogenic lineages, as evidenced by enhanced transmissibility and pathogenicity, arises mainly from two mechanisms: the horizontal acquisition of exogenous virulence determinants via mobile genetic elements, and the regulatory adaptation of endogenous genes to optimize pathogenic potential. Created in BioRender (https://BioRender.com/vo79j4j).

A paradigm shift has emerged that disarms pathogens by neutralizing virulence determinants without imposing lethal selective pressure. Virulence factors contribute to pathogenicity through colonization, immune evasion, and host tissue damage (Jiang et al., 2023). Anti-virulence therapy targets these factors or their regulatory pathways (Li et al., 2024), inhibiting adhesion (Lau et al., 2023), disrupting effector transport, and attenuating collective behavior (Nadeem et al., 2020). Virulence factors are traditionally categorized into several groups, such as adhesion factors (Lau et al., 2023), invasion factors (Hotinger et al., 2021), toxins, effector delivery systems, immune modulators, biofilm-associated factors (Flemming et al., 2023; Subramani et al., 2025), motility, regulation, and nutritional or metabolic factors, as shown in **Figure 3**. The regulatory systems governing these factors are complex, with finely tuned signaling cascades (Stebbins and Galán, 2003; Choi and Choi, 2022; Liu et al., 2022a; Qin et al., 2022). Since virulence factors typically do not participate in fundamental growth processes (Totsika, 2017), this approach can significantly reduce the selective pressure on bacteria, thereby limiting the rapid evolution of resistance (Totsika, 2017). It also exhibits high selectivity, which may preserve the host’s microbiota in some cases, but this is not guaranteed for all anti-virulence strategies, such as quorum-quenching (QQ) enzymes. Furthermore, anti-virulence therapy can act synergistically with antibiotics, reducing the required antibiotic dosage while prolonging efficacy (Totsika, 2017).

However, this strategy faces inherent limitations that are often underappreciated. First, pathogens encode multiple redundant virulence factors, making it impractical for a single agent to completely block all pathogenic processes; thus, combination regimens are generally required. Second, because the therapy does not directly kill bacteria, ultimate pathogen clearance remains highly dependent on the host’s immune function (Dickey et al., 2017). Consequently, it cannot rapidly reduce bacterial loads in immunocompromised patients with confirmed severe infections, leading to potentially insufficient efficacy or delayed onset of action. Third, pathogens may persistently colonize the host after treatment discontinuation, causing latent damage (Dickey et al., 2017), or regain pathogenicity through adaptive evolution that circumvents the targeted factors (Allen et al., 2014; Totsika, 2017).

### 2.4 Comparative analysis and clinical implications

Having reviewed each category, we now present a critical comparative analysis. The three axes are not equally viable as universal solutions; they occupy distinct positions within a tripartite trade-off involving response time, host dependency, and evolutionary sustainability. Harnessing host factors (vaccines, therapeutic antibodies and host-directed therapies) excel in durability and specificity but are fundamentally constrained by host biological variables (immunocompetence for vaccines and antibodies; infection-stage timing for HDTs), in addition to extended development cycles for vaccines (Zheng et al., 2025; Hotchkiss and Monneret, 2026). Direct killing strategies (phages and AMPs) offer the fastest onset but create strong selective bottlenecks, making them evolutionarily vulnerable. Anti-virulence strategies, by contrast, impose minimal fitness costs, thereby stabilizing the pathogen’s genome and limiting resistance emergence (Totsika, 2017). However, they sacrifice the speed of bacterial clearance and require precise diagnostic pre-identification of the targeted virulence factor.

This dissection reveals a crucial insight: no single strategy can simultaneously satisfy the demands of acute therapy, broad patient coverage, and resistance evasion. We argue that the common practice of presenting these modalities as competing alternatives is conceptually flawed. Instead, their optimal deployment is inherently sequential and context dependent. For fulminant infections, direct killing agents are indispensable. For chronic colonization or post-acute relapse prevention, anti-virulence agents offer an ideal maintenance modality by preserving the microbiome and preventing disease progression without provoking resistance. In the specific context of hospital-acquired and biofilm-associated infections, where the patient’s immune status is often compromised and repeated antibiotic courses are common, the moderate clearance rate of anti-virulence therapy is offset by its exceptional safety profile and resistance resilience.

Among the various virulence regulatory networks, the QS system is acknowledged as the most extensively studied regulatory hub governing the expression of diverse pathogenic cascades (Ruan et al., 2026). Crucially, QS inhibition distinguishes itself from anti-toxin or anti-adhesion approaches that target a single structural factor. QS acts as a master switch that concurrently modulates biofilm maturation, toxin secretion, and immune evasion. This hierarchical control position endows QS inhibitors with a broader spectrum of action compared to agents targeting individual downstream factors. Nevertheless, given the intricate cross-talk and regulatory redundancy among QS circuits, complete abrogation of virulence production remains challenging with a single inhibitor. Given that QS serves as a central regulatory hub for bacterial virulence, and that quorum sensing inhibitors (QSIs) represent one of the most intensively investigated anti-virulence modalities, the following section provides a comprehensive survey of this class of agents.

## 3 Focusing on anti-virulence agents targeting QS

### 3.1 Fundamental concepts and mechanisms of QS

QS enables bacterial cell-to-cell communication through the production, detection, and response to signaling molecules, known as autoinducers (Mukherjee and Bassler, 2019). The widespread existence of this intercellular communication system among bacteria in nature has been well documented (Waters and Bassler, 2005; Subramani et al., 2025). QS can regulate bacterial bioluminescence and govern biofilm development, virulence expression, motile behaviors, and stress responses (Ogawara, 2021). QS allows bacteria to dynamically regulate gene expression in response to population density, particularly during infection. This system helps bacteria coordinate the expression of virulence factors to evade host immunity and enhance invasiveness. Under low-density conditions, QS typically suppresses the expression of virulence factors to avoid premature detection by the host immune system. At high densities, it relieves this suppression and promotes large-scale expression to facilitate rapid host attack. This “latent-burst” strategy allows bacteria to optimize the timing and efficacy of the infection. Moreover, QS can modulate host immune responses by suppressing inflammatory signals or enhancing bacterial survival within host cells (Fan et al., 2022). By synchronizing group behaviors and individual behaviors, QS significantly improves the adaptability of bacterial communities to environmental changes. Key mechanisms include the sequential expression of virulence factors, division of labor among subpopulations in invasion and immune evasion, and the formation of persister cells, all of which contribute to more efficient and sustained infections (Striednig and Hilbi, 2022). Due to its complexity and plasticity, QS enables pathogens to adaptively adjust their pathogenic strategies in response to environmental changes (Letizia et al., 2025).

The QS system consists of four main components: autoinducers as signaling molecules, autoinducer synthases for biosynthesis, cognate autoinducer receptors for signal detection and binding, and downstream signal transduction components that convert autoinducer-mediated signals into alterations in gene expression (Mukherjee and Bassler, 2019). As the bacterial density increases, autoinducers accumulate in the extracellular environment. When their concentration reaches the QS threshold, autoinducers bind to intracellular receptors, forming complexes that modulate the transcription of related genes. The expression and secretion of virulence factors in many bacterial pathogens of plants, animals, and humans are controlled by QS, making it a critical system for regulating virulence factors.

**Figure 4:**
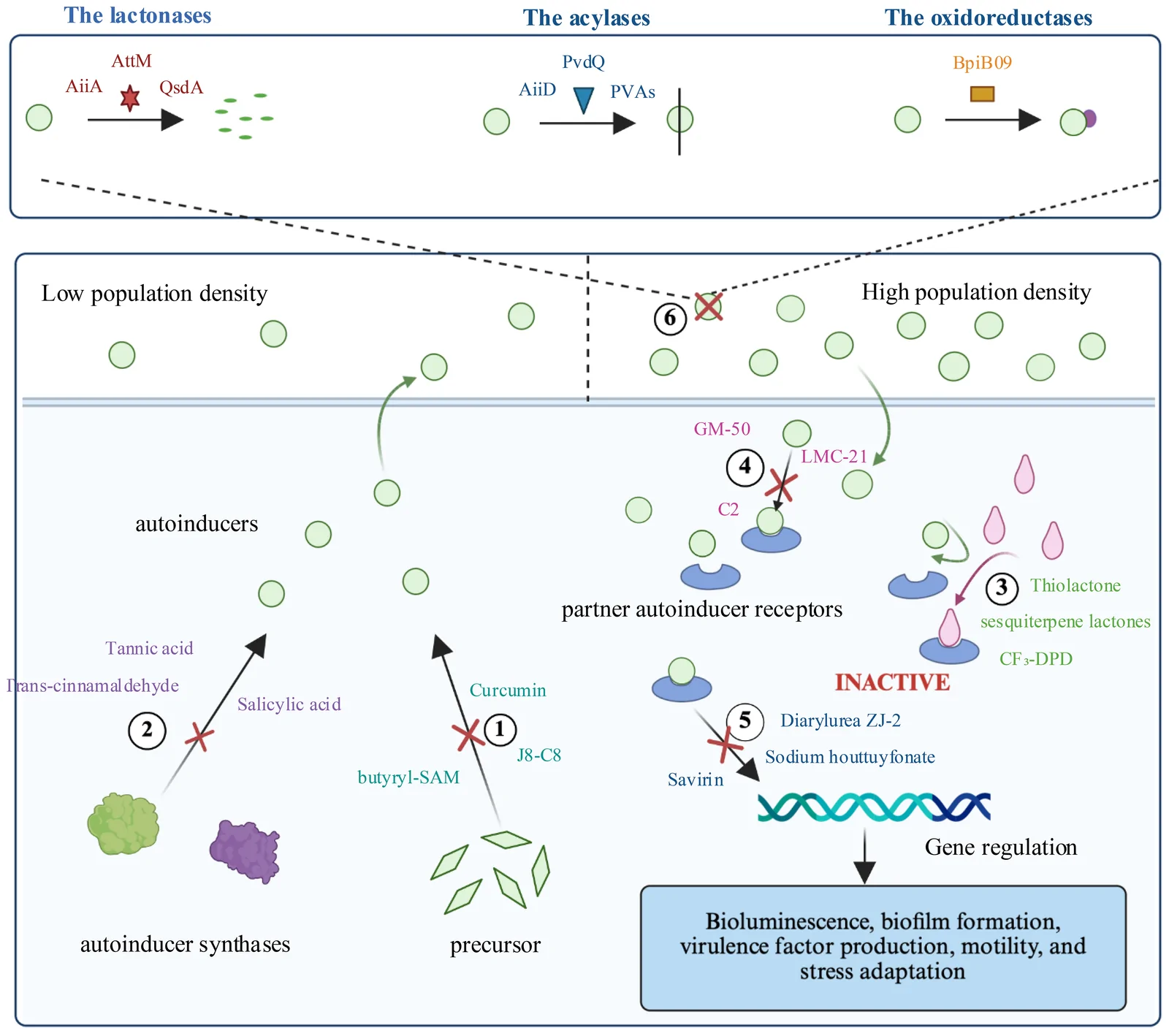
Mechanisms of action of QSIs and QQ enzymes as anti-virulence agents inhibiting QS. ①-⑤ Mechanisms of action of QSIs: ① Blocking the production of QS signaling molecules by interfering with the synthesis of precursor signaling molecules. ② Blocking the production of QS signaling molecules by inhibiting the activity of key synthases. ③ Interfering with signal transduction by competitively binding to receptor proteins using signaling molecule analogs. ④ Impeding the binding of native QS signaling molecules to receptor proteins and disrupting the normal conformation of receptors, thereby inhibiting QS system function. ⑤ Inhibiting transcription factors by preventing their binding to DNA, which suppresses the expression of downstream virulence genes. ⑥ Mechanisms of QQ enzymes: degrading or chemically modifying QS signaling molecules, thereby reducing their concentration below the threshold required to activate QS. Created in BioRender (https://BioRender.com/eiiip2l).

Owing to the central role of QS in bacterial metabolism, virulence, and resistance, disrupting this system is an anti-virulence strategy with significant therapeutic potential (Lu et al., 2022). Anti-virulence agents targeting QS represent a novel approach to combat bacterial infections, particularly drug-resistant infections, as an alternative to traditional antibiotics. Unlike conventional antibiotics, these agents modulate the behavior and population dynamics of bacterial populations and attenuate pathogenicity without directly killing bacteria or interfering with their essential life processes. This class of compounds includes QSIs and QQ enzymes. QSIs are primarily small molecules that target multiple steps in the signaling cascade, whereas QQ enzymes are biocatalysts that degrade or modify signaling molecules, thereby preventing bacteria from detecting these signals. The mechanism by which QSIs and QQ enzymes target the QS system is shown in **Figure 4**.

### 3.2 QSIs: diverse mechanisms of action

QSIs are small-molecule compounds that disrupt bacterial QS systems. They attenuate the expression of virulence factors by interfering with the synthesis, release, perception, or downstream signaling of QS signals (Spaggiari et al., 2025). Mechanistically, QSIs disrupt bacterial communication through the following primary modes of action (**Table 1**).

**Table 1:** Mechanism-based classification of quorum sensing inhibitors (QSIs).

Mechanism type	Examples	Target bacteria	Function	Reference
Inhibition of precursor synthesis	triclosan	*P. aeruginosa*	Inhibits acyl-ACP biosynthesis, suppressing AHL synthesis.	(Grandclément et al., 2016)
	butyryl-SAM, sine- fungin	*P. aeruginosa*, *Agrobacterium tumefaciens*	SAM analogs competitively inhibit LuxI-type AHL synthases.	(Grandclément et al., 2016)
	J8-C8	*A. tumefaciens*	Competitively inhibits the acyl-ACP carrier, thereby suppressing AHL biosynthesis	(Grandclément et al., 2016)
	Curcumin	*Bacillus cereus*	Can bind to LuxS protein, competitively displace SRH, and inhibit AI-2 synthesis	(Wang et al., 2025)
Inhibition of synthetic enzyme activity	Salicylic acid	*P. aeruginosa*	Interferes with AHL signaling, reduces virulence factor expression	(Chang et al., 2014)
	Tannic acid	*P. aeruginosa*	Specifically inhibits short-chain AHL synthase RhlI	(Chang et al., 2014)
	Trans-cinnamaldehyde	*P. aeruginosa*, *Vibrio spp*	Inhibits RhlI by binding its active site	(Chang et al., 2014)
	IAA	*Burkholderia mallei*	inhibits BmaI1 acyl-HSL synthase activity	(Christensen et al., 2013)
	SXH (16)	*Bacillus subtilis*	directly inhibit the LuxS enzyme, thereby blocking the production of DPD	(Wnuk et al., 2009)
Inhibition of gene transcription	Sodium houttuyfonate	*P. aeruginosa*	Dose-dependently inhibits AHL production by downregulating lasI	(Wu et al., 2014)
	Mangifera indica L.	*Chromobacterium violaceum*, *P. aeruginosa*, *Aeromonas hydrophila*	Directly inhibits las-controlled transcription	(Husain et al., 2017)
	Savirin	*S. aureus*	Selectively inhibits the transcriptional regulator AgrA of the agr system, preventing its binding to DNA	(Sully et al., 2014)
	Diarylurea ZJ-2	*Enterococcus faecium*	inhibits peptidoglycan hydrolase and adhesion gene expression	(Xie et al., 2022)
	novel indolin-2-one derivative	*E. faecalis*	It inhibits the fsr QS system	(Alzahrani et al., 2022)
Inhibition of receptor proteins	GM-50	*P. aeruginosa*	competes with C4HSL for binding to the receptor	(Bernabè et al., 2022)
	LMC-21	*Vibrio species*	interfere with AI-2 binding to the LuxPQ complex	(Brackman et al., 2009)
	C2	*P. aeruginosa*	activates an inhibitory receptor within the QS system, namely the LuxR-type receptor protein QscR	(Weng et al., 2014)

Some targets are also addressed by signal molecule analogues, which are presented in **Table 2**.

#### 3.2.1 Inhibition of signaling molecule production

QSIs suppress the synthesis of signaling molecules by interfering with precursor synthesis or enzymatic activity. *N*-acyl homoserine lactone (AHL) autoinducers are synthesized from the essential metabolic precursors S-adenosylmethionine (SAM) and acylated acyl carrier protein (acyl-ACP). As both SAM and acyl-ACP play crucial roles in bacterial survival, complete inhibition of their biosynthesis would kill bacteria, creating selective pressure. Therefore, a strategic approach at this stage requires precise and specific inhibition. For example, structural analogs of SAM, such as butyryl-SAM and sinefungin, act as competitive inhibitors of LuxI-type AHL synthases, specifically attenuating signaling molecule production without affecting cellular SAM biosynthesis or concentration (Grandclément et al., 2016). trans-Cinnamaldehyde specifically inhibits the short-chain AHL synthase RhlI. This compound binds to the substrate-binding site of the AHL synthase, competitively blocking substrate binding and inhibiting AHL synthesis (Chang et al., 2014).

Autoinducer-2 (AI-2) is a signaling molecule synthesized by the LuxS enzyme, a key enzyme in the activated methyl cycle that participates in SAM metabolism to generate 4,5-dihydroxy-2,3-pentanedione (DPD). DPD spontaneously cyclizes in water to form distinct isomers with AI-2 activity. These isomers can interconvert in solution, enabling AI-2 to transmit signals between different bacterial species (Pereira et al., 2013). Curcumin can form hydrogen bonds with the LuxS protein of *Bacillus cereus*, competitively displacing S-ribosyl homocysteine (SRH), leading to SRH accumulation and inhibition of AI-2 synthesis (Wang et al., 2025).

#### 3.2.2 Disruption of signal transduction

In bacterial QS systems, three main types of autoinducers act as signaling molecules: AHL, autoinducing peptide (AIP) and AI-2. The differences among these autoinducers directly determine their specific binding patterns to their respective receptors. AHL and AIP primarily mediate intraspecies communication, whereas AI-2 is responsible for interspecies communication (Rutherford and Bassler, 2012; Wu et al., 2020). QSIs that disrupt signal transduction include structural analogs of these autoinducers (**Table 2**), such as AHL, AIP, and AI-2 analogs, which competitively bind to receptor proteins and block signaling.

**Table 2:** Autoinducer analogues as quorum sensing inhibitors (QSIs).

Mechanism type	Examples	Target bacteria	Function	Reference
AHL analogs	pyrazole and pyrazolo[1,5-a]pyrimidine derivatives	multidrug-resistant Gram-positive and Gram-negative bacteria	the signal diffusion was decreased	(Ragab et al., 2023)
	sesquiterpene lactones	*P. aeruginosa*	inhibition of AHLs production	(Amaya et al., 2012)
	Carvacrol,Eugenol	multiple bacterial species	Reducing production of AHLs	(Bouyahya et al., 2022)
	Y-31	*P. aeruginosa*	Mimic natural signaling molecules as competitive antagonists	(Yan et al., 2024)
	psoralen	*P. aeruginosa*		(Wen et al., 2024)
	Thiolactone	Gram-negative bacteria		(McInnis and Blackwell, 2011)
	Dihydrofuranone derivatives	*P. aeruginosa* and *Burkholderia cenocepacia*		(Brackman et al., 2012)
	lactams,solenopsin A alkaloid,isothiocyanate iberin	*P. aeruginosa*		(Grandclément et al., 2016)
	OZDO	*P. aeruginosa*	interfered with three core regulatory proteins	(Wu et al., 2025)
AIP analogs	WS9326A, WS9326B and cochinmicin II/III	*S. aureus*, *E. faecalis*, *Clostridium perfringens*	competitively binds to histidine kinase receptors of AIPs	(Desouky et al., 2015)
	Truncated or “tail-less” derivative, Peptoids	*S. aureus*	Interferes with AIP-AgrC binding	(Gorske and Blackwell, 2006)
	trAIP-II, N3A AIP-II, AIP-II Lactam/Lactone	*S. aureus*	compete with AIPs for the same binding site on the receptor AgrC	(Lyon et al., 2002)
AI-2 analogs	Alkyl-DPD Analogues	*Vibrio harveyi*, *Salmonella typhimurium*	blocking the signal recognition step	(Lowery et al., 2009)
	CF_3_-DPD	Multiple bacterial species	Binds to the AI-2 receptor and competitively inhibits natural AI-2 binding	(Pereira et al., 2013)
	Isobutyl-DPD, Cyclopentyl-DPD	*E. coli*, *Salmonella typhimurium*	Phosphorylated by LsrK, then inhibits LsrR.	(Gamby et al., 2012)
	Phenyl-DPD, Heptyl-DPD	*P. aeruginosa*	Phenomenological, mechanism unknown	(Gamby et al., 2012)
Other signal molecules analogs	ZBzl-YAA591	*E. faecalis*	Inhibits AIP-receptor binding	(Nakayama et al., 2013)

QS, quorum sensing; QSI, quorum sensing inhibitor; AHL, acyl-homoserine lactone; AIP, autoinducing peptide; AI-2, autoinducer-2; DPD, 4,5-dihydroxy-2,3-pentanedione.

AHLs are primarily found in Gram-negative bacteria and consist of a homoserine lactone (HSL) ring and a variable fatty acid side chain. The length of the side chain and the substitution at the C3 position determine their specificities. Short-chain AHLs diffuse freely into the cell, whereas long-chain AHLs require active transport. Once inside, they bind to LuxR family receptors. The lactone ring and the amide bond form hydrogen bonds with conserved residues on intracellular LuxR family receptors, while the side chains fit into hydrophobic pockets, inducing receptor folding and dimerization, which in turn leads to DNA binding and transcriptional regulation. Variations in side-chain length and substituents provide the structural basis for intraspecific specificity (Ampomah-Wireko et al., 2021; Liu et al., 2022b). Modifications to AHL analogs primarily target the homoserine lactone ring, acyl side chain, or both. A series of analogs was designed and synthesized by replacing the amide bond in native AHL molecules with a triazole ring. Among these, derivatives bearing long alkyl chains or aromatic substituents have proven to be effective QSIs. These compounds inhibit QS by competitively binding to AHL receptors. They structurally mimic native AHLs but do not activate, or only partially activate, QS responses, thereby blocking the expression of downstream genes (Brackman et al., 2012). In silico molecular docking and virtual screening of 37 oxazolidinone-derived compounds, followed by experimental validation, demonstrated that OZDO competitively inhibits the binding of QS signal molecules to their receptor proteins, thereby effectively blocking signal perception within the QS pathway and significantly suppressing virulence factor production (Wu et al., 2025).

AIP is primarily found in Gram-positive bacteria. It is a processed and modified oligopeptide that contains an internal thiolactone bond at the C-terminus, forming a macrocyclic core, along with an additional variable exocyclic tail (Tal-Gan et al., 2013). This structure binds specifically to membrane-bound histidine kinase AgrC or other two-component sensors through two key features: its macrocyclic thiolactone core, which presents a triangular hydrophobic knob, and the insertion of the exocyclic tail into the receptor’s binding cleft. The sequence diversity of the cyclic core and the tail determines strong intraspecific recognition and cross-inhibition effects. AIP must be transported out of the cell via a dedicated transporter such as AgrB. Once outside, it binds to the two-component histidine kinase sensor on the cell membrane, inducing a conformational change that activates intracellular kinase activity. This change then transmits the signal through a phosphorylation cascade to the response regulator (McBrayer et al., 2020; Gless et al., 2025). The cyclodepsipeptides WS9326A, WS9326B, and Cochimnicin II/III, which are naturally produced by actinomycetes, competitively bind to the histidine kinase receptors of AIPs. By occupying ligand-binding sites, they prevent native AIPs from binding to their cognate receptors, thereby inhibiting QS signal transduction. This results in the suppressed activation of virulence genes and effective quenching of QS in various Gram-positive bacterial pathogens (Nakayama et al., 2013).

AI-2, a cross-species signal, is derived from the precursor DPD, which is produced from SRH by the conserved enzyme LuxS. DPD can cyclize into different furanose derivatives depending on bacterial species and environmental conditions, such as borate availability. Different bacteria use specific receptors such as LuxP, which binds the boron-containing form, and LsrB, which binds the boron-free form, to recognize distinct AI-2 conformations, leading to signal transduction or internalization. Because LuxS is highly conserved across many bacteria, AI-2 has been proposed as a universal signal for interbacterial communication (Chen et al., 2002; Zhao et al., 2018). A diverse array of structural AI-2 analogs has been synthesized using organic synthetic approaches. The most common design strategy involves modifying the C1 methyl group by introducing linear, branched, or cyclic alkyl substituents or a trifluoromethyl group. These analogs may act as agonists or antagonists across different bacterial species, reflecting the structural diversity of AI-2 receptors among different organisms. These modifications aim to interfere with receptor binding by altering the terminal chemical moiety of the signaling molecule. Modifications at the C5 position have also been explored, where the stereochemical configuration significantly affects the binding affinities of LuxP and LsrB. Additionally, naturally occurring QSIs, including brominated furanones and cinnamaldehyde, have been identified, however, their activity is generally not specific to AI-2-mediated signaling but instead exerts broader inhibitory effects (Pereira et al., 2013). A series of cyclic and aromatic AI-2 analogs was synthesized using the established diazocarbonyl aldol reaction method. Among these, analogs such as cyclopentyl-DPD significantly inhibited QS in *E. coli*. In contrast, aromatic analogs exhibited minimal effects on *E. coli* and Salmonella typhimurium but effectively suppressed pyocyanin production in *P. aeruginosa* (Gamby et al., 2012).

#### 3.2.3 Inhibition of gene transcription

Certain QSIs function by modulating specific signaling pathways, such as repressing gene transcription or interfering with transcription factors. For example, through high-throughput screening, Savirin was identified as a compound that selectively inhibits the transcriptional regulator AgrA within the agr system. It prevents AgrA from binding to DNA, thereby suppressing the expression of downstream virulence genes and specifically inhibiting the agr system in *S. aureus* (Sully et al., 2014). Mango leaf extracts inhibit the production of virulence factors and biofilm formation in diverse pathogenic bacteria by disrupting QS-mediated transcriptional activation (Husain et al., 2017).

#### 3.2.4 Inhibition of the binding of signaling molecules to receptor proteins

Inhibition strategies targeting receptor proteins involve suppressing their biosynthesis, disrupting their proper assembly, or competitively or non-competitively blocking the binding of QS autoinducers to their homologous receptors. For instance, LMC-21 and its analogues specifically disrupt the LuxPQ receptor complex within the AI-2-mediated QS pathway (Brackman et al., 2009). A phenolic derivative, GM-50, acts primarily by competitively binding to the RhlR receptor, inhibiting its activity, and subsequently downregulating the production of multiple virulence factors in *P. aeruginosa* (Bernabè et al., 2022). Another notable example is C2, which in contrast to conventional QSIs, functions as an exogenous signal molecule that activates the QS Control Repressor, a LuxR-type receptor. This activation indirectly suppresses the entire QS network of *P. aeruginosa* (Weng et al., 2014).

#### 3.2.5 Advantages and limitations of QSIs

QSIs offer multiple advantages as potential therapeutic agents. First, QSIs selectively suppress the expression of virulence factors without interfering with bacterial growth or essential protein synthesis (Gutiérrez-Barranquero et al., 2015; Patel et al., 2023). By specifically interfering with the synthesis, detection, or degradation of autoinducer signals, this approach reduces selection pressure on fundamental bacterial metabolism, substantially lowering the likelihood of resistance development while minimizing the risk of host microbiome disruption (Rasko and Sperandio, 2010; Panda et al., 2022; Chong et al., 2025). Second, QSIs demonstrate excellent resensitizing and synergistic effects with certain antibiotics; for instance, by disrupting biofilm integrity and suppressing efflux pump activity, they facilitate antibiotic penetration, thereby enabling dose reduction and preventing the emergence of resistant strains (Grandclément et al., 2016; Wen et al., 2024). Furthermore, QSIs are widely sourced from natural products and synthetic compounds, exhibiting structural diversity that provides a rich pool of candidates for broad-spectrum anti-pathogenic agents (Odularu et al., 2022). These properties collectively make QSIs an ideal therapeutic option against infections caused by multidrug-resistant bacteria, particularly highly virulent and resistant strains such as *P. aeruginosa* and *S. aureus* (Spaggiari et al., 2025).

Despite these theoretical advantages, to date, no *de novo* molecule specifically developed as a QSIs has successfully entered Phase I/II clinical trials, and the vast majority of reported QSIs remain at the preclinical stage (Spaggiari et al., 2025; Truong-Thanh et al., 2025). Although a few compounds have reached clinical evaluation, they were not registered as QSIs per se; rather, they are approved drugs whose QSIs activity was identified retrospectively. Azithromycin is a representative example, having been investigated as a quorum-sensing blocker for the prevention of *P. aeruginosa a* ventilator-associated pneumonia (van Delden et al., 2012); however, the trial was prematurely terminated owing to discontinuation of financial support. Beyond this translational void, the development of QSIs is further constrained by several interconnected biological, pharmacological, and clinical limitations. Biologically, although QSIs target a central hub, they typically suppress only a subset of QS-regulated genes; due to extensive crosstalk and redundancy among multiple QS circuits (e.g., Las, Rhl, and PQS in *P. aeruginosa*), inhibiting a single system often fails to completely abrogate virulence production (Patel et al., 2023; Wen et al., 2024; Chong et al., 2025). Given the redundancy of QS circuits, single-target QSIs are insufficient to fully block virulence. Multitarget strategies, either single molecules engaging multiple QS receptors or rational combinations of complementary inhibitors, can overcome this limitation. They reduce compensatory resistance but pose pharmacokinetic and safety challenges. Recent combination approaches with antibiotics and phages show promise (Yang et al., 2026). Pharmacologically, many QSIs exhibit poor stability, rapid *in vivo* clearance, low bioavailability, and insufficient tissue penetration, complicating the assessment of their effective concentrations and undermining *in vivo* efficacy (Patel et al., 2023; Nowak et al., 2026). Clinically, their antimicrobial efficacy remains partially dependent on host immune function, leading to variable outcomes; for immunocompromised patients, such as those in intensive care, QSIs monotherapy may be ineffective, typically necessitating combination with conventional antibiotics (Yuan et al., 2022; Ran et al., 2025). Additionally, certain QSIs may only be effective against either acute or chronic infections, but not both simultaneously, reflecting their restricted scope, as QS differentially regulates virulence factor burst in acute infections versus biofilm and persister cell formation in chronic infections (Rutherford and Bassler, 2012; LaSarre and Federle, 2013). From a safety perspective, certain QSIs may induce adverse reactions or toxicity, and in some instances, they may inadvertently select for hypervirulent strains or drive bacterial adaptive evolution, challenging their long-term utility (Brackman et al., 2011; Grandclément et al., 2016).

The risk of QSI-driven resistance, while real, warrants a more nuanced interpretation. Early *in vitro* studies demonstrated that bacteria such as *P. aeruginosa* can develop resistance to QSIs, for example, brominated furanone C-30, through efflux pump mutations (Maeda et al., 2012). However, recent *in vivo* evolution studies have refined this concern by revealing that QSI-resistant cells spread substantially slower during host infection, approximately 10-fold slower than antibiotic resistance, when initially rare (Yang and Defoirdt, 2024; Defoirdt, 2025). Moreover, because QS often controls public good traits, for example, extracellular proteases, resistant “cheaters” are exploited by sensitive cells, imposing a fitness cost that further restricts resistance dissemination (Defoirdt, 2025). These findings do not dismiss the resistance risk but indicate that QSIs may still offer a more evolutionarily sustainable strategy than traditional antibiotics (Sikdar and Elias, 2022; Guo et al., 2024).

Encouragingly, the pharmacokinetic and physicochemical hurdles are being actively overcome through innovative formulation strategies. Advanced nanocarriers, such as pH-responsive nanoparticles, polymer–QSI conjugates, and nanostructured lipid carriers, significantly enhance solubility, protect against enzymatic degradation, and enable targeted, sustained release within the biofilm microenvironment, thereby improving local bioavailability and penetration (Chen et al., 2023; Soukarieh et al., 2023; Tang et al., 2026). Furthermore, combining QSIs with conventional antibiotics achieves synergistic effects that reduce required antibiotic dosages, concurrently mitigating toxicity and selection pressure. Collectively, these advances position QSI-based therapies as a practically feasible and evolutionarily sustainable strategy against biofilm-associated and multidrug-resistant infections, provided that rigorous pharmacokinetic optimization and rational combination regimens are integrated into their clinical development.

### 3.3 QQ enzymes: biocatalytic degradation of signals

#### 3.3.1 Classification and sources of QQ enzymes

To counteract the adverse effects of the QS system, QQ enzymes have evolved. QQ enzymes interrupt QS signaling by modifying, disrupting, or degrading QS signal molecules, thereby reducing their concentrations below the threshold required to trigger QS (Dong et al., 2000). QQ enzymes interfere with QS signaling pathways through multiple mechanisms and exhibit diverse structures. Three major classes have been characterized: (1) Lactonases: hydrolyze the lactone ring of AHLs, inactivating the signal (Zhang et al., 2002). Among the lactonases, AHL-lactonases exhibit the most pronounced variations in sequence and structure. The catalytic mechanism of lactonases involves metal ion-mediated hydrolysis; however, the specific mechanisms vary slightly among different families (Elias and Tawfik, 2012). (2) Acylases: act on AHLs by cleaving their amide bonds, thereby generating a fatty acid and a homoserine lactone. They exhibit a characteristic Ntn-hydrolase fold and catalyze reactions via a covalently bound intermediate (Bokhove et al., 2010). (3) Oxidoreductases: degrade AHLs via oxidoreduction reactions. BpiB09 is a novel short-chain dehydrogenase/reductase identified in a soil metagenome. As an NADP-dependent oxidoreductase, it attenuates bacterial virulence by chemically reducing the key QS signal molecule 3-oxo-C_12_-HSL in *P. aeruginosa*, converting it into a 3-hydroxy derivative (Bijtenhoorn et al., 2011). The classification of QQ enzyme types, along with representative examples and their sources, is summarized in **Table 3**.

**Table 3:** Classification, sources, substrates, and target pathogens of quorum-quenching (QQ) enzymes.

Enzyme type	Examples	Source	Target	Target pathogen	Reference
Lactonase	AiiA	*Bacillus* sp. *240B1*	AHLs	*Erwinia carotovora*	(Dong et al., 2000)
	AttM	*Agrobacterium tumefaciens*	AHLs	*Agrobacterium tumefaciens*	(Zhang et al., 2002)
	QsdA	*Rhodococcus erythropolis*	AHLs	*Agrobacterium tumefaciens*, etc.	(Uroz et al., 2008)
	AhlD	*Arthrobacter* sp.	AHLs	*Erwinia carotovora*	(Park et al., 2003)
	SsoPox	*Sulfolobus solfataricus*	AHLs	*P.aeruginosa*	(Guendouze et al., 2017)
	PONs	mammals	AHLs	*P.aeruginosa*	(Yang et al., 2005)
	AidH	*Ochrobactrum* sp.	AHLs	*Pseudomonas fluorescens 2P24*, *Pectobacterium carotovorum subsp. carotovorum Z3-3*	(Mei et al., 2010)
	LrsL	*Labrenzia* sp. *VG12*	AHLs	*P.aeruginosa*	(Rehman et al., 2022)
	AidC	*Chryseobacterium* sp. *StRB126*	AHLs	Gram-negative bacteria	(Wang et al., 2012)
	MomL	*Muricauda olearia*	AHLs	Gram-negative bacteria	(Tang and Zhang, 2018)
	GcL	*Parageobacillus caldoxylosilyticus*	AHLs	Gram-negative bacteria	(Corbella et al., 2024)
	AaL	*Alicyclobacter acidoterrestris*	AHLs	Gram-negative bacteria	(Bergonzi et al., 2017)
	JydB	*Rhodococcus* sp. *BH4*	AHLs	*Aeromonas* sp. *T3-4*	(Ryu et al., 2020)
Acylase	AiiD	*Ralstonia strain XJ12B*	AHLs	*P.aeruginosa*	(Lin et al., 2003)
	AhlD	*Arthrobacter sp*	AHLs	*Erwinia carotovora*	(Park et al., 2003)
	Porcine kidney acylase I	porcine kidney	AHLs	*P.aeruginosa* and *Microbacterium imperiale*	(Xu et al., 2003)
	PvdQ	*P. aeruginosa*	AHLs	*P.aeruginosa* and *Burkholderia cenocepacia*	(Liu et al., 2019)
	QuiP	*P. aeruginosa*	AHLs	*P.aeruginosa* and Ralstonia	(Huang et al., 2006)
	PVAs	*Pectobacterium atrosepticum, Agrobacterium tumefaciens*	AHLs	*P.aeruginosa*	(Sunder et al., 2017)
	AiiC	*Anabaena* sp. *PC C 7120*	AHLs	Gram-negative bacteria	(Romero et al., 2008)
	AlbP	*Brucella melitensis*	AHLs	*Brucella melitensis*	(Terwagne et al., 2013)
	AmiE	*Acinetobacter* sp. *Strain Ooi24*	AHLs	*P.aeruginosa*	(Ochiai et al., 2014)
	QsdB	Uncultured bacterium (metagenome)	AHLs	*E. coli* and *Pectobacterium*	(Tannières et al., 2013)
Oxidoreductases	BpiB09	*Uncultured bacterium (metagenome)*	AHLs	*P.aeruginosa*	(Bijtenhoorn et al., 2011)

QQ enzymes are diverse in type and are found in bacteria, archaea, eukaryotes, and mammals. For example, Quip is an acylase encoded by the PA1032 gene in *P. aeruginosa* and is classified as an endogenous enzyme (Huang et al., 2006). SsoPox is derived from the extremely thermophilic archaeon *Sulfolobus solfataricus*, which thrives in high-temperature and acidic environments. Consequently, this enzyme exhibits exceptional thermostability and chemical stability (Lin et al., 2003). Derived from the gram-negative plant pathogens *Pectobacterium atrosepticum* and *Agrobacterium tumefaciens*, the PVAs are acyltransferase class enzymes that degrade long-chain AHLs (Sunder et al., 2017). Porcine kidney acylase I is derived from the porcine kidney and belongs to the acylase class. It exhibits optimal activity at pH 10 and 76 °C and can reduce biofilm formation (Xu et al., 2003).

#### 3.3.2 Advantages and limitations of QQ enzymes

QQ enzymes can degrade various AHLs, including those with different acyl chain lengths. This broad substrate specificity may enable them to interfere with the QS systems of various bacteria. Some QQ enzymes exhibit high catalytic efficiency. They can hydrolyze lactone substrates through multiple mechanisms (Corbella et al., 2024). Furthermore, QQ enzymes can degrade various QS signaling molecules, including but not limited to AHLs. By degrading these signaling molecules, QQ enzymes can selectively inhibit the expression of virulence factors in pathogenic bacteria, without directly killing the bacteria and thus reducing the risk of drug resistance. In agriculture and aquaculture, some QQ enzymes can replace antibiotics, reducing environmental residues and avoiding disruption of microbial communities (Grandclément et al., 2016). These enzymes play important roles in microbial competition and host defense and hold promise for various applications.

Despite these promising attributes, no QQ enzyme has yet advanced into human clinical trials, several limitations have been observed in the performance of QQ enzymes. Some QQ enzymes are prone to inactivation in complex environments and require chemical modification or carrier optimization to improve stability (Fan et al., 2022). The activity of QQ enzymes may be regulated by host metabolites, thereby affecting their actual efficacy. Certain QQ enzymes may interfere with the beneficial QS of symbiotic bacteria, thereby disrupting the balance of the microbial community. The large-scale production of high-purity QQ enzymes is costly, and delivery efficiency issues need to be addressed (Zhang et al., 2023; Corbella et al., 2024).

## 4 Conclusions and perspectives

According to the latest assessment by the WHO, only a handful of clinical-stage antibiotics targeting priority pathogens have demonstrated true innovation, with non-traditional alternatives accounting for approximately 40% of antimicrobial products in development (World Health Organization, 2025). However, current progress remains insufficient to address the escalating threat of AMR (Sati et al., 2025). Given the inherent trade-offs among host-factor-based, direct-killing, and anti-virulence strategies delineated in this review, we posit that no single modality will completely “replace” traditional antibiotics, just as antibiotics were never a single solution to infectious diseases (Upadhayay et al., 2023). The real hope lies not in a mythical “next-generation antibiotic”, but in a paradigm shift: from a “warfare model” that directly eliminates pathogens to a “disruption and neutralization model” that disarms their pathogenic capabilities. This necessitates the establishment of an intelligent defense system integrating multiple precise measures, transitioning from “sterilization” to “microbial control”.

In this transition, artificial intelligence (AI) is emerging as an indispensable accelerator. Beyond the biological strategies discussed above, AI-driven virtual screening, molecular docking, and generative design are poised to overcome the translational bottlenecks that have long plagued alternative anti-infectives (Melo et al., 2021). Specifically, for QS-targeted anti-virulence therapy, tools such as Dockey (a protein-ligand docking tool) (Du et al., 2023) can efficiently evaluate the binding affinities of billions of compounds against QS receptors, while generative models like SyntheMol have already achieved notable *in vitro* hit rates against multidrug-resistant *Acinetobacter baumannii* (Swanson et al., 2024) and have generated lead compounds with distinct mechanisms of action against Neisseria gonorrhoeae and *Staphylococcus aureus* (Krishnan et al., 2025). Furthermore, structure-based optimization leveraging AlphaFold and molecular dynamics simulations can refine these hit-to-lead candidates (Zheng et al., 2026), while AI-driven QSAR and machine learning classifiers (Sanapalli et al., 2026; Zhong et al., 2026) offer predictive insights into physicochemical properties, metabolic stability, and tissue penetration, parameters that critically influence the *in vivo* efficacy of QSIs and QQ enzymes.

Looking ahead, the convergence of AI with multi-omics, synthetic biology, and automated high-throughput screening holds the potential to herald an era of data-driven precision anti-infective strategies (Melo et al., 2021). By accelerating hit discovery, de-risking pharmacokinetic failures, and enabling the rational design of synergistic combination regimens, AI could serve as a powerful enabling tool. Ultimately, rather than supplanting the biological strategies surveyed in this review, AI provides the computational scaffolding necessary to realize their full potential, bringing us closer to the integrated, multi-pronged defense system that could represent a viable way forward in the post-antibiotic era.
